# 5,11,17,23-Tetra-*tert*-butyl-25,26,27,28-tetra­propyn­yloxy-2,8,14,20-tetra­thia­calix[4]arene

**DOI:** 10.1107/S1600536809043992

**Published:** 2009-11-07

**Authors:** Xiong Li, Hong-Wei Han, Xiang-Gao Meng

**Affiliations:** a Wuhan National Laboratory for Optoelectronics, Huazhong University of Science and Technology, Wuhan 430074, People’s Republic of China; b College of Chemistry, Huazhong Normal University, Wuhan 430079, People’s Republic of China

## Abstract

The title compound [systematic name: 5,11,17,23-tetra-*tert*-butyl-25,26,27,28-tetra­propyn­yloxy-2,8,14,20-tetra­thia­calix[4]arene], C_52_H_56_O_4_S_4_, is an alkyl­ated product bearing four propyne groups at the lower rim of a 5,11,17,23-tetra-*tert*-butyl-tetrathia­calix[4]arene. The mol­ecule is located on a crystallographic twofold rotation axis, running through two S atoms and perpendicular to the long axis of the mol­ecule. The four propyne groups, located in an alternate fashion above and below the mean plane of the four S atoms, are almost parallel to the calixarene long axis. The dihedral angle between the two crystallographically independent benzene rings is 86.77 (14)°. Two *tert*-butyl groups are disordered over two positions with site occupancies of 0.59 (2) and 0.41 (2).

## Related literature

For related structures, see: Kumagai & Hasegawa (1997 ▶); Kasyan *et al.* (2007 ▶).
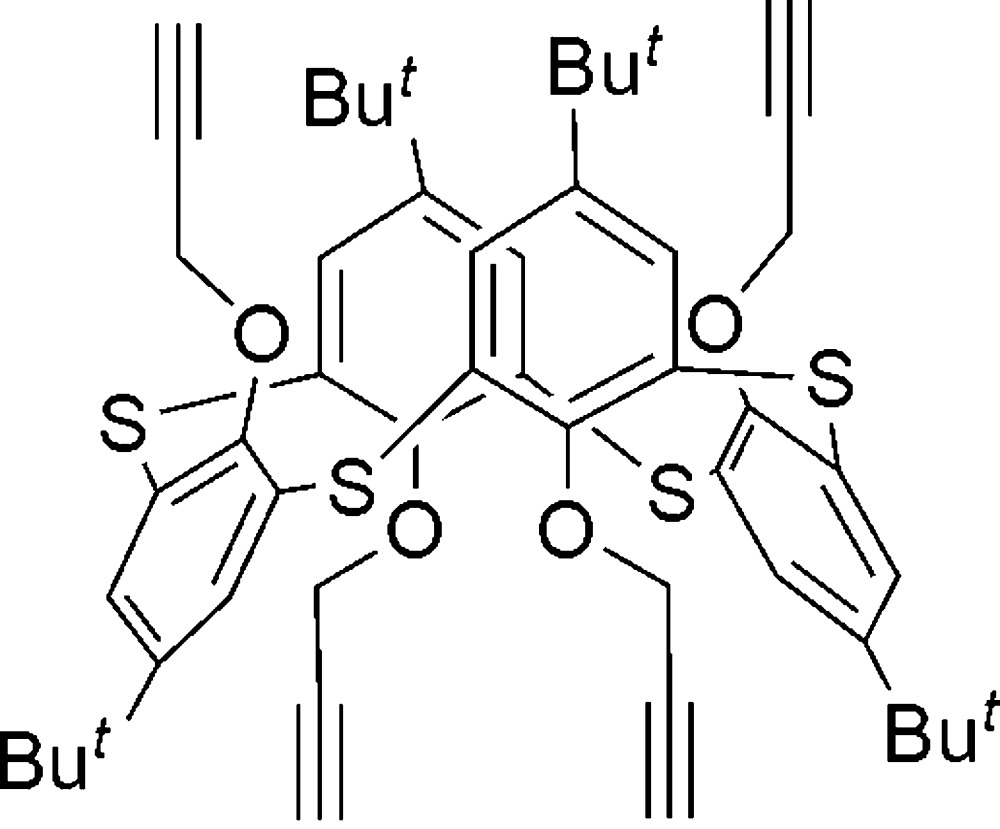


## Experimental

### 

#### Crystal data

C_52_H_56_O_4_S_4_
*M* *_r_* = 873.21
Monoclinic, 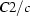

*a* = 13.5662 (11) Å
*b* = 19.1815 (16) Å
*c* = 18.1595 (15) Å
β = 90.398 (1)°
*V* = 4725.4 (7) Å^3^
*Z* = 4
Mo *K*α radiation
μ = 0.25 mm^−1^
*T* = 150 K
0.20 × 0.10 × 0.10 mm

#### Data collection

Bruker SMART APEX CCD area-detector diffractometer
Absorption correction: none
26256 measured reflections
5115 independent reflections
4543 reflections with *I* > 2σ(*I*)
*R*_int_ = 0.028

#### Refinement

*R*[*F* ^2^ > 2σ(*F* ^2^)] = 0.043
*wR*(*F* ^2^) = 0.130
*S* = 1.06
5115 reflections
310 parameters
39 restraints
H-atom parameters constrained
Δρ_max_ = 0.38 e Å^−3^
Δρ_min_ = −0.19 e Å^−3^

### 

Data collection: *SMART* (Bruker, 1997 ▶); cell refinement: *SAINT* (Bruker, 1997 ▶); data reduction: *SAINT*; program(s) used to solve structure: *SHELXS97* (Sheldrick, 2008 ▶); program(s) used to refine structure: *SHELXL97* (Sheldrick, 2008 ▶); molecular graphics: *SHELXTL* (Sheldrick, 2008 ▶); software used to prepare material for publication: *SHELXTL*.

## Supplementary Material

Crystal structure: contains datablocks global, I. DOI: 10.1107/S1600536809043992/is2455sup1.cif

Structure factors: contains datablocks I. DOI: 10.1107/S1600536809043992/is2455Isup2.hkl

Additional supplementary materials:  crystallographic information; 3D view; checkCIF report

## Figures and Tables

**Fig. 1.**
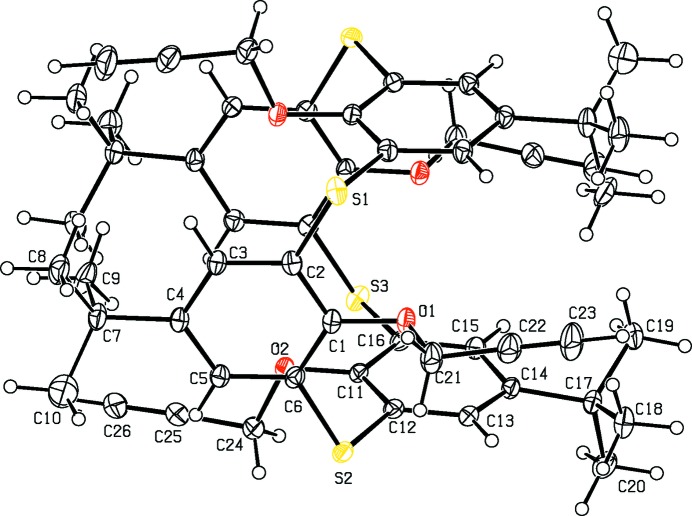
View of the molecule of (I), showing the atom-labelling scheme. Displacement ellipsoids are drawn at the 50% probability level. One of the tert-butyl group attached to the phenyl rings was found to be disordered over two positions and only one part of disordered groups has been drawn for clarity.

## supplementary crystallographic information

### Comment

The title compound, (I), possessing four propyne groups on the lower rim of 1,3-alternate thiacalix[4]arene backbone in the solid state, represents a novel example of flexible thiacalix[4]arene derivative, whose ^1^H NMR spectrum revealed the simultaneous presence of the partial cone (PC) and 1,3-alternate (1,3-A) conformers in 1:2 ratio at room temperature. It was prepared by the base-promoted condensation of the *p*-*tert*-butyl-thiacalixarene with 3-bromoprop-1-yne.

The single crystal of C_52_H_56_O_4_S_4_ was obtained by recrystallization from its chloroform–acetone (1:1 v/v) solution. It crystallises in the monoclinic system (space group C2/c) and there is no solvent molecule present in the lattice. The size of the thiacalixarene cavity could be defined by the distances of these sulfur atoms to the centroid formed by the four sulfur atoms, which are in the range of 3.667–3.996 Å.

The observed C—S distance, in the range of 1.777–1.781 Å, is in agreement with the one observed for the parent *p*-*tert*-butyl-thiacalixarene (1.785 Å) (Kumagai *et al.*, 1997). For the ether junction between the calix and the pendent arms, the carbon to oxygen distances are in the range of 1.376–1.378 Å for C(Ph)—O and 1.432–1.449 Å for O—C(propyne). The four propyne groups are located in an alternate fashion below and above the main plane of the thiacalixarene. The CH_2_CCH fragments are almost linear with CCC angle of 177.41 and 177.56°. The pendent arms bearing the acetylene groups are oriented almost parallel to the calix long axis. The aromatic moieties on the same face of the molecule are almost parallel. It is worth noting that because of the short nature of the spacer connecting the acetylene group to the calix, the acetylene (CCH) groups are in close proximity with the *tert*-butyl moieties located on the same face (distance in the range of ca. 3.46–4.55 Å).

### Experimental

The title compound has been prepared by a direct base-promoted condensation reaction of thiacalixarene with an excess of 3-bromoprop-1-yne. A solution of *p*-*tert*-butyl-thiacalixarene (0.5 mmol) and 3-bromoprop-1-yne (5 mmol) in dry acetone (25 ml) was refluxed for 24 h in the presence of Na_2_CO_3_. The reaction mixture was separated by column chromatography on silica gel with chloroform–petroleum ether as eluant. Single crystals of (I) were grown from a chloroform–acetone (1:1 v/v) solution and was stable in air. ^1^H NMR study revealed that the powder was composed of the title compound in both PC and 1,3-A conformations in a 1:2 ratio in CDCl_3_ solution at room temperature. Spectroscopic analysis: ^1^H NMR (CDCl_3_, δ, p.p.m.): 1.08 (s, 18H, PC), 1.27 (s, 36H, 1,3-A.), 1.31 (s, 9H, PC), 1.43 (s, 9H, PC), 2.19 (s, 1H, PC), 2.43 (s, 4H, 1,3-A.), 2.53 (s, 3H, PC),4.50 (s, 2H, PC), 4.69 (d, 8H, J =2.1Hz, 1,3-A.), 4.79 (s, 6H, PC), 7.05 (d, 2H, J = 2.1 Hz, PC), 7.53 (s, 2H,, PC), 7.58 (s, 8H, 1,3-A); ; 7.90 (s, 2H, PC); analysis, calculated for C_52_H_56_O_4_S_4_: C 71.52, H 6.46, S 14.69%; found: C 71.37, H 6.40, S 14.62%.

### Refinement

All H atoms were initially located in a difference Fourier map. The methyl H atoms were then constrained to an ideal geometry with C—H distances of 0.98 Å and *U*_iso_(H) = 1.5*U*_eq_(C), but each group was allowed to rotate freely about its C—C bond. All other H atoms were placed in geometrically idealized positions and constrained to ride on their parent atoms with C—H distances in the range 0.95–0.99 Å and *U*_iso_(H) = 1.2*U*_eq_(C). One of the *tert*-butyl groups of each thiacalixarene molecule is disordered over two positions with site occupancies of 0.59 (2) and 0.41 (2), resulting in bond distances that deviate from ideal values. The restraints on the C—C bond distance (SADI) and on displacement parameters (ISOR) were applied for the disordered *tert*-butyl group. The crystal used was a partial twin; the twin matrix was (100/010/001). The fraction of the minor domain was refined to 0.0531 (7).

### Crystal data

**Table:**

C_52_H_56_O_4_S_4_	*F*(000) = 1856
*M**_r_* = 873.21	*D*_x_ = 1.227 Mg m^−^^3^
Monoclinic, *C*2/*c*	Mo *K*α radiation, λ = 0.71073 Å
Hall symbol: -C 2yc	Cell parameters from 9940 reflections
*a* = 13.5662 (11) Å	θ = 2.2–28.3°
*b* = 19.1815 (16) Å	µ = 0.25 mm^−^^1^
*c* = 18.1595 (15) Å	*T* = 150 K
β = 90.398 (1)°	Block, colorless
*V* = 4725.4 (7) Å^3^	0.20 × 0.10 × 0.10 mm
*Z* = 4	

### Data collection

**Table:**

Bruker SMART APEX CCD area-detector diffractometer	4543 reflections with *I* > 2σ(*I*)
Radiation source: fine focus sealed Siemens Mo tube	*R*_int_ = 0.028
graphite	θ_max_ = 27.0°, θ_min_ = 1.1°
0.3° wide ω exposures scans	*h* = −17→15
26256 measured reflections	*k* = −24→24
5115 independent reflections	*l* = −23→23

### Refinement

**Table:**

Refinement on *F*^2^	Primary atom site location: structure-invariant direct methods
Least-squares matrix: full	Secondary atom site location: difference Fourier map
*R*[*F*^2^ > 2σ(*F*^2^)] = 0.043	Hydrogen site location: inferred from neighbouring sites
*wR*(*F*^2^) = 0.130	H-atom parameters constrained
*S* = 1.06	*w* = 1/[σ^2^(*F*_o_^2^) + (0.0816*P*)^2^ + 2.5666*P*] where *P* = (*F*_o_^2^ + 2*F*_c_^2^)/3
5115 reflections	(Δ/σ)_max_ = 0.002
310 parameters	Δρ_max_ = 0.38 e Å^−^^3^
39 restraints	Δρ_min_ = −0.19 e Å^−^^3^

### Special details

**Table:**

Geometry. All esds (except the esd in the dihedral angle between two l.s. planes) are estimated using the full covariance matrix. The cell esds are taken into account individually in the estimation of esds in distances, angles and torsion angles; correlations between esds in cell parameters are only used when they are defined by crystal symmetry. An approximate (isotropic) treatment of cell esds is used for estimating esds involving l.s. planes.
Refinement. Refinement of F^2^ against ALL reflections. The weighted R-factor wR and goodness of fit S are based on F^2^, conventional R-factors R are based on F, with F set to zero for negative F^2^. The threshold expression of F^2^ > 2sigma(F^2^) is used only for calculating R-factors(gt) etc. and is not relevant to the choice of reflections for refinement. R-factors based on F^2^ are statistically about twice as large as those based on F, and R- factors based on ALL data will be even larger.

### Fractional atomic coordinates and isotropic or equivalent isotropic displacement parameters (Å^2^)

**Table:**

	*x*	*y*	*z*	*U*_iso_*/*U*_eq_	Occ. (<1)
C1	0.48682 (12)	0.76830 (9)	0.14123 (9)	0.0244 (3)	
C2	0.43521 (13)	0.72657 (8)	0.19086 (9)	0.0248 (3)	
C3	0.33290 (13)	0.72817 (8)	0.19175 (10)	0.0250 (3)	
H3	0.2988	0.6999	0.2261	0.030*	
C4	0.27892 (13)	0.77027 (8)	0.14345 (9)	0.0241 (3)	
C5	0.33161 (13)	0.81294 (9)	0.09602 (9)	0.0251 (3)	
H5	0.2967	0.8431	0.0636	0.030*	
C6	0.43391 (13)	0.81261 (9)	0.09481 (9)	0.0248 (3)	
C7	0.16650 (13)	0.77476 (10)	0.14820 (11)	0.0318 (4)	
C8	0.1182 (5)	0.7075 (3)	0.1744 (7)	0.0491 (19)	0.59 (2)
H8A	0.1436	0.6955	0.2234	0.074*	0.59 (2)
H8B	0.1334	0.6698	0.1399	0.074*	0.59 (2)
H8C	0.0467	0.7140	0.1766	0.074*	0.59 (2)
C9	0.1460 (6)	0.8308 (4)	0.2071 (4)	0.0514 (19)	0.59 (2)
H9A	0.0747	0.8376	0.2116	0.077*	0.59 (2)
H9B	0.1769	0.8748	0.1925	0.077*	0.59 (2)
H9C	0.1733	0.8156	0.2545	0.077*	0.59 (2)
C10	0.1194 (11)	0.7981 (8)	0.0760 (5)	0.055 (3)	0.59 (2)
H10A	0.0477	0.8001	0.0816	0.083*	0.59 (2)
H10B	0.1360	0.7649	0.0370	0.083*	0.59 (2)
H10C	0.1443	0.8445	0.0629	0.083*	0.59 (2)
C11	0.50988 (12)	0.96579 (8)	0.14291 (9)	0.0224 (3)	
C12	0.56075 (13)	0.92436 (9)	0.09304 (9)	0.0239 (3)	
C13	0.66255 (13)	0.92995 (9)	0.08692 (9)	0.0241 (3)	
H13	0.6960	0.9012	0.0526	0.029*	
C14	0.71670 (12)	0.97671 (9)	0.12982 (9)	0.0231 (3)	
C15	0.66507 (13)	1.01550 (9)	0.18251 (9)	0.0243 (3)	
H15	0.7004	1.0460	0.2143	0.029*	
C16	0.56335 (13)	1.01026 (8)	0.18924 (9)	0.0232 (3)	
C17	0.82759 (13)	0.98826 (10)	0.11823 (10)	0.0276 (4)	
C18	0.87520 (15)	0.92647 (11)	0.07943 (13)	0.0395 (5)	
H18A	0.8647	0.8841	0.1084	0.059*	
H18B	0.9461	0.9349	0.0744	0.059*	
H18C	0.8454	0.9207	0.0305	0.059*	
C19	0.88093 (15)	1.00021 (14)	0.19170 (12)	0.0432 (5)	
H19A	0.8561	1.0430	0.2146	0.065*	
H19B	0.9519	1.0048	0.1831	0.065*	
H19C	0.8689	0.9605	0.2245	0.065*	
C20	0.83994 (16)	1.05423 (11)	0.07084 (12)	0.0388 (5)	
H20A	0.8068	1.0475	0.0233	0.058*	
H20B	0.9102	1.0630	0.0629	0.058*	
H20C	0.8107	1.0942	0.0962	0.058*	
C21	0.62688 (15)	0.71823 (12)	0.08857 (12)	0.0418 (5)	
H21A	0.6097	0.7331	0.0379	0.050*	
H21B	0.5977	0.6717	0.0971	0.050*	
C22	0.73363 (16)	0.71483 (12)	0.09731 (13)	0.0439 (5)	
C23	0.81945 (18)	0.70994 (15)	0.10290 (16)	0.0587 (7)	
H23	0.8890	0.7060	0.1074	0.070*	
C24	0.36289 (14)	1.01176 (11)	0.09750 (11)	0.0363 (5)	
H24A	0.3718	0.9956	0.0462	0.044*	
H24B	0.3943	1.0581	0.1026	0.044*	
C25	0.25865 (15)	1.01698 (11)	0.11376 (10)	0.0372 (4)	
C26	0.17363 (17)	1.02289 (16)	0.12435 (13)	0.0557 (7)	
H26	0.1050	1.0277	0.1329	0.067*	
O1	0.58854 (9)	0.76737 (7)	0.14086 (7)	0.0292 (3)	
O2	0.40865 (8)	0.96324 (6)	0.14765 (6)	0.0253 (3)	
S1	0.5000	0.66803 (3)	0.2500	0.02986 (16)	
S2	0.49578 (3)	0.86815 (2)	0.03174 (2)	0.02872 (14)	
S3	0.5000	1.06748 (3)	0.2500	0.02795 (16)	
C8'	0.1289 (8)	0.6988 (4)	0.1405 (11)	0.057 (3)	0.41 (2)
H8'1	0.1543	0.6708	0.1816	0.085*	0.41 (2)
H8'2	0.1520	0.6791	0.0940	0.085*	0.41 (2)
H8'3	0.0567	0.6985	0.1412	0.085*	0.41 (2)
C9'	0.1361 (14)	0.8091 (11)	0.2190 (6)	0.085 (5)	0.41 (2)
H9'1	0.0661	0.8215	0.2162	0.128*	0.41 (2)
H9'2	0.1754	0.8513	0.2269	0.128*	0.41 (2)
H9'3	0.1469	0.7768	0.2601	0.128*	0.41 (2)
C10'	0.1209 (12)	0.8110 (9)	0.0812 (7)	0.032 (2)	0.41 (2)
H10D	0.0491	0.8060	0.0825	0.048*	0.41 (2)
H10E	0.1462	0.7896	0.0361	0.048*	0.41 (2)
H10F	0.1383	0.8606	0.0820	0.048*	0.41 (2)

### Atomic displacement parameters (Å^2^)

**Table:**

	*U*^11^	*U*^22^	*U*^33^	*U*^12^	*U*^13^	*U*^23^
C1	0.0181 (8)	0.0254 (8)	0.0297 (8)	−0.0020 (6)	0.0019 (6)	−0.0092 (6)
C2	0.0221 (8)	0.0204 (8)	0.0319 (8)	−0.0015 (6)	−0.0004 (6)	−0.0048 (6)
C3	0.0214 (8)	0.0205 (8)	0.0332 (9)	−0.0037 (6)	0.0025 (7)	−0.0022 (6)
C4	0.0210 (8)	0.0199 (8)	0.0314 (8)	−0.0029 (6)	0.0012 (6)	−0.0055 (6)
C5	0.0223 (9)	0.0250 (8)	0.0281 (8)	−0.0025 (6)	0.0000 (6)	−0.0031 (6)
C6	0.0232 (9)	0.0274 (9)	0.0239 (8)	−0.0064 (7)	0.0038 (6)	−0.0058 (6)
C7	0.0197 (9)	0.0307 (9)	0.0449 (10)	−0.0021 (7)	0.0036 (7)	0.0073 (8)
C8	0.025 (2)	0.042 (3)	0.080 (5)	−0.0074 (17)	0.006 (3)	0.028 (3)
C9	0.032 (3)	0.067 (4)	0.056 (3)	−0.001 (2)	0.023 (2)	−0.015 (3)
C10	0.035 (4)	0.076 (8)	0.055 (4)	−0.006 (4)	−0.001 (3)	0.001 (3)
C11	0.0194 (8)	0.0240 (8)	0.0240 (8)	−0.0022 (6)	0.0029 (6)	0.0057 (6)
C12	0.0241 (9)	0.0262 (8)	0.0213 (7)	−0.0050 (6)	0.0012 (6)	0.0023 (6)
C13	0.0234 (9)	0.0273 (9)	0.0218 (7)	−0.0024 (6)	0.0036 (6)	0.0018 (6)
C14	0.0190 (8)	0.0251 (8)	0.0253 (8)	−0.0023 (6)	0.0013 (6)	0.0049 (6)
C15	0.0246 (9)	0.0215 (8)	0.0268 (8)	−0.0037 (6)	0.0004 (7)	0.0014 (6)
C16	0.0256 (9)	0.0189 (8)	0.0251 (8)	−0.0001 (6)	0.0052 (6)	0.0031 (6)
C17	0.0204 (9)	0.0344 (10)	0.0280 (8)	−0.0053 (7)	0.0024 (7)	0.0004 (7)
C18	0.0238 (10)	0.0415 (11)	0.0531 (12)	0.0001 (8)	0.0050 (8)	−0.0054 (9)
C19	0.0247 (10)	0.0694 (15)	0.0356 (10)	−0.0110 (9)	−0.0030 (8)	0.0000 (10)
C20	0.0334 (11)	0.0405 (11)	0.0425 (11)	−0.0113 (8)	0.0095 (8)	0.0067 (9)
C21	0.0270 (10)	0.0521 (13)	0.0464 (11)	0.0055 (9)	0.0068 (8)	−0.0188 (10)
C22	0.0341 (12)	0.0503 (13)	0.0475 (12)	0.0055 (9)	0.0112 (9)	−0.0090 (10)
C23	0.0291 (12)	0.0713 (18)	0.0759 (18)	0.0093 (11)	0.0086 (11)	−0.0144 (14)
C24	0.0261 (10)	0.0478 (12)	0.0350 (10)	0.0047 (8)	0.0002 (8)	0.0158 (8)
C25	0.0314 (11)	0.0510 (12)	0.0292 (9)	0.0058 (9)	−0.0025 (8)	0.0082 (8)
C26	0.0304 (12)	0.095 (2)	0.0414 (12)	0.0149 (12)	0.0018 (9)	0.0193 (12)
O1	0.0174 (6)	0.0349 (7)	0.0355 (7)	−0.0009 (5)	0.0024 (5)	−0.0089 (5)
O2	0.0189 (6)	0.0302 (6)	0.0268 (6)	−0.0001 (5)	0.0016 (5)	0.0069 (5)
S1	0.0241 (3)	0.0191 (3)	0.0463 (4)	0.000	−0.0038 (3)	0.000
S2	0.0245 (2)	0.0405 (3)	0.0213 (2)	−0.01046 (17)	0.00285 (17)	−0.00272 (16)
S3	0.0301 (3)	0.0176 (3)	0.0362 (3)	0.000	0.0093 (3)	0.000
C8'	0.019 (3)	0.058 (4)	0.093 (7)	−0.011 (3)	−0.012 (4)	0.041 (4)
C9'	0.055 (6)	0.139 (9)	0.064 (5)	0.028 (7)	0.027 (4)	0.012 (6)
C10'	0.010 (4)	0.030 (4)	0.055 (5)	0.002 (2)	−0.005 (3)	0.018 (3)

### Geometric parameters (Å, °)

**Table:**

C1—O1	1.380 (2)	C15—H15	0.9500
C1—C6	1.393 (2)	C16—S3	1.7815 (17)
C1—C2	1.397 (2)	C17—C18	1.525 (3)
C2—C3	1.389 (2)	C17—C19	1.531 (3)
C2—S1	1.7815 (18)	C17—C20	1.540 (3)
C3—C4	1.396 (2)	C18—H18A	0.9800
C3—H3	0.9500	C18—H18B	0.9800
C4—C5	1.390 (2)	C18—H18C	0.9800
C4—C7	1.531 (2)	C19—H19A	0.9800
C5—C6	1.388 (2)	C19—H19B	0.9800
C5—H5	0.9500	C19—H19C	0.9800
C6—S2	1.7790 (17)	C20—H20A	0.9800
C7—C9'	1.505 (8)	C20—H20B	0.9800
C7—C10	1.522 (6)	C20—H20C	0.9800
C7—C8	1.524 (5)	C21—O1	1.438 (2)
C7—C10'	1.528 (6)	C21—C22	1.457 (3)
C7—C9	1.543 (5)	C21—H21A	0.9900
C7—C8'	1.549 (7)	C21—H21B	0.9900
C8—H8A	0.9800	C22—C23	1.172 (3)
C8—H8B	0.9800	C23—H23	0.9500
C8—H8C	0.9800	C24—O2	1.440 (2)
C9—H9A	0.9800	C24—C25	1.450 (3)
C9—H9B	0.9800	C24—H24A	0.9900
C9—H9C	0.9800	C24—H24B	0.9900
C10—H10A	0.9800	C25—C26	1.176 (3)
C10—H10B	0.9800	C26—H26	0.9500
C10—H10C	0.9800	S1—C2^i^	1.7815 (18)
C11—O2	1.377 (2)	S3—C16^i^	1.7815 (17)
C11—C12	1.392 (2)	C8'—H8'1	0.9800
C11—C16	1.398 (2)	C8'—H8'2	0.9800
C12—C13	1.390 (2)	C8'—H8'3	0.9800
C12—S2	1.7790 (17)	C9'—H9'1	0.9800
C13—C14	1.394 (2)	C9'—H9'2	0.9800
C13—H13	0.9500	C9'—H9'3	0.9800
C14—C15	1.403 (2)	C10'—H10D	0.9800
C14—C17	1.536 (2)	C10'—H10E	0.9800
C15—C16	1.390 (2)	C10'—H10F	0.9800
			
O1—C1—C6	121.07 (15)	C18—C17—C20	109.41 (16)
O1—C1—C2	120.09 (15)	C19—C17—C20	108.15 (16)
C6—C1—C2	118.78 (15)	C14—C17—C20	107.78 (15)
C3—C2—C1	120.03 (16)	C17—C18—H18A	109.5
C3—C2—S1	119.73 (13)	C17—C18—H18B	109.5
C1—C2—S1	120.17 (13)	H18A—C18—H18B	109.5
C2—C3—C4	121.70 (16)	C17—C18—H18C	109.5
C2—C3—H3	119.1	H18A—C18—H18C	109.5
C4—C3—H3	119.1	H18B—C18—H18C	109.5
C5—C4—C3	117.41 (16)	C17—C19—H19A	109.5
C5—C4—C7	121.24 (16)	C17—C19—H19B	109.5
C3—C4—C7	121.05 (15)	H19A—C19—H19B	109.5
C6—C5—C4	121.70 (17)	C17—C19—H19C	109.5
C6—C5—H5	119.1	H19A—C19—H19C	109.5
C4—C5—H5	119.1	H19B—C19—H19C	109.5
C5—C6—C1	120.30 (16)	C17—C20—H20A	109.5
C5—C6—S2	118.91 (14)	C17—C20—H20B	109.5
C1—C6—S2	120.78 (13)	H20A—C20—H20B	109.5
C10—C7—C8	109.8 (7)	C17—C20—H20C	109.5
C9'—C7—C10'	111.7 (10)	H20A—C20—H20C	109.5
C9'—C7—C4	110.6 (8)	H20B—C20—H20C	109.5
C10—C7—C4	112.4 (6)	O1—C21—C22	108.74 (17)
C8—C7—C4	113.6 (3)	O1—C21—H21A	109.9
C10'—C7—C4	112.2 (7)	C22—C21—H21A	109.9
C10—C7—C9	108.3 (7)	O1—C21—H21B	109.9
C8—C7—C9	107.1 (4)	C22—C21—H21B	109.9
C4—C7—C9	105.3 (4)	H21A—C21—H21B	108.3
C9'—C7—C8'	113.3 (7)	C23—C22—C21	177.6 (3)
C10'—C7—C8'	103.0 (8)	C22—C23—H23	180.0
C4—C7—C8'	105.6 (5)	O2—C24—C25	109.43 (15)
C7—C8—H8A	109.5	O2—C24—H24A	109.8
C7—C8—H8B	109.5	C25—C24—H24A	109.8
C7—C8—H8C	109.5	O2—C24—H24B	109.8
C7—C9—H9A	109.5	C25—C24—H24B	109.8
C7—C9—H9B	109.5	H24A—C24—H24B	108.2
C7—C9—H9C	109.5	C26—C25—C24	177.2 (2)
C7—C10—H10A	109.5	C25—C26—H26	180.0
C7—C10—H10B	109.5	C1—O1—C21	112.21 (13)
C7—C10—H10C	109.5	C11—O2—C24	111.29 (13)
O2—C11—C12	121.30 (14)	C2—S1—C2^i^	101.86 (11)
O2—C11—C16	119.83 (15)	C6—S2—C12	101.19 (7)
C12—C11—C16	118.86 (15)	C16—S3—C16^i^	103.93 (11)
C13—C12—C11	120.35 (15)	C7—C8'—H8'1	109.5
C13—C12—S2	119.00 (13)	C7—C8'—H8'2	109.5
C11—C12—S2	120.50 (13)	H8'1—C8'—H8'2	109.5
C12—C13—C14	121.66 (16)	C7—C8'—H8'3	109.5
C12—C13—H13	119.2	H8'1—C8'—H8'3	109.5
C14—C13—H13	119.2	H8'2—C8'—H8'3	109.5
C13—C14—C15	117.30 (15)	C7—C9'—H9'1	109.5
C13—C14—C17	121.93 (15)	C7—C9'—H9'2	109.5
C15—C14—C17	120.71 (15)	H9'1—C9'—H9'2	109.5
C16—C15—C14	121.48 (15)	C7—C9'—H9'3	109.5
C16—C15—H15	119.3	H9'1—C9'—H9'3	109.5
C14—C15—H15	119.3	H9'2—C9'—H9'3	109.5
C15—C16—C11	120.17 (15)	C7—C10'—H10D	109.5
C15—C16—S3	119.57 (13)	C7—C10'—H10E	109.5
C11—C16—S3	119.89 (13)	H10D—C10'—H10E	109.5
C18—C17—C19	108.66 (17)	C7—C10'—H10F	109.5
C18—C17—C14	111.68 (15)	H10D—C10'—H10F	109.5
C19—C17—C14	111.07 (15)	H10E—C10'—H10F	109.5
			
O1—C1—C2—C3	179.25 (14)	C11—C12—C13—C14	−0.1 (2)
C6—C1—C2—C3	1.9 (2)	S2—C12—C13—C14	175.43 (13)
O1—C1—C2—S1	−3.8 (2)	C12—C13—C14—C15	3.3 (2)
C6—C1—C2—S1	178.85 (12)	C12—C13—C14—C17	−174.00 (15)
C1—C2—C3—C4	0.6 (3)	C13—C14—C15—C16	−3.2 (2)
S1—C2—C3—C4	−176.28 (13)	C17—C14—C15—C16	174.18 (15)
C2—C3—C4—C5	−2.5 (2)	C14—C15—C16—C11	−0.2 (2)
C2—C3—C4—C7	−176.29 (15)	C14—C15—C16—S3	−173.19 (13)
C3—C4—C5—C6	1.8 (2)	O2—C11—C16—C15	−177.05 (14)
C7—C4—C5—C6	175.61 (16)	C12—C11—C16—C15	3.5 (2)
C4—C5—C6—C1	0.7 (3)	O2—C11—C16—S3	−4.1 (2)
C4—C5—C6—S2	179.33 (13)	C12—C11—C16—S3	176.46 (12)
O1—C1—C6—C5	−179.88 (14)	C13—C14—C17—C18	−22.1 (2)
C2—C1—C6—C5	−2.6 (2)	C15—C14—C17—C18	160.62 (16)
O1—C1—C6—S2	1.5 (2)	C13—C14—C17—C19	−143.60 (18)
C2—C1—C6—S2	178.80 (12)	C15—C14—C17—C19	39.1 (2)
C5—C4—C7—C9'	−107.1 (9)	C13—C14—C17—C20	98.09 (19)
C3—C4—C7—C9'	66.5 (9)	C15—C14—C17—C20	−79.2 (2)
C5—C4—C7—C10	29.2 (7)	C6—C1—O1—C21	−89.5 (2)
C3—C4—C7—C10	−157.3 (7)	C2—C1—O1—C21	93.3 (2)
C5—C4—C7—C8	154.7 (6)	C22—C21—O1—C1	−173.78 (17)
C3—C4—C7—C8	−31.8 (6)	C12—C11—O2—C24	−88.73 (19)
C5—C4—C7—C10'	18.5 (8)	C16—C11—O2—C24	91.80 (18)
C3—C4—C7—C10'	−168.0 (8)	C25—C24—O2—C11	−169.36 (16)
C5—C4—C7—C9	−88.5 (4)	C3—C2—S1—C2^i^	−115.53 (15)
C3—C4—C7—C9	85.0 (4)	C1—C2—S1—C2^i^	67.56 (12)
C5—C4—C7—C8'	130.0 (7)	C5—C6—S2—C12	116.61 (14)
C3—C4—C7—C8'	−56.5 (7)	C1—C6—S2—C12	−64.77 (15)
O2—C11—C12—C13	177.19 (14)	C13—C12—S2—C6	123.77 (14)
C16—C11—C12—C13	−3.3 (2)	C11—C12—S2—C6	−60.69 (15)
O2—C11—C12—S2	1.7 (2)	C15—C16—S3—C16^i^	−123.33 (16)
C16—C11—C12—S2	−178.81 (12)	C11—C16—S3—C16^i^	63.63 (12)

Symmetry codes: (i) −*x*+1, *y*, −*z*+1/2.
